# Efficacy of mesenchymal stromal cells for the treatment of knee osteoarthritis: a meta-analysis of randomized controlled trials

**DOI:** 10.1186/s13018-020-02128-0

**Published:** 2021-01-06

**Authors:** Huazheng Qu, Shui Sun

**Affiliations:** 1 Shandong Provincial Hospital, Cheeloo College of Medicine, Shandong University, Jinan, 250021 Shandong China; 2 Department of Joint Surgery, the Third Hospital of Jinan, Jinan, 250132 China; 3 Department of Joint Surgery, Shandong Provincial Hospital Affiliated to Shandong University, Jinan, 250021 China

**Keywords:** Mesenchymal stromal cells, Knee osteoarthritis, Meta-analysis

## Abstract

**Background:**

Mesenchymal stromal cells (MSCs) are used as an emerging new option for the treatment of knee osteoarthritis (OA). However, their efficacy remains controversial across studies with different doses of MSCs and cell processing methods. We conducted this meta-analysis to assess the efficacy of MSCs in the treatment of knee OA.

**Methods:**

Randomized controlled trials (RCTs) published in PubMed, Embase, Web of Science, SinoMed (Chinese BioMedical Literature Service System, China), and CNKI (National Knowledge Infrastructure, China) databases were systematically reviewed. The pain level and function improvements were evaluated using visual analog scale (VAS), McMaster Universities Osteoarthritis Index (WOMAC), and International Knee Documentation Committee (IKDC). The pooled estimate was calculated with weighted mean difference (WMD) with 95% confidence intervals (95%CIs).

**Results:**

Nine RCTs involving 476 patients were included in this meta-analysis. The pooled estimate showed that the treatment of MSCs significantly reduced VAS, WOMAC pain, WOMAC stiffness, and WOMAC function scores at a long-term follow-up (12 or 24 months). However, for the IKDC and WOMAC total scores, MSCs also showed significant improvement in these outcomes, although this was not statistically significant when compared to the control.

**Conclusion:**

Based on the current studies, our results suggested that MSCs were a promising option for the treatment of patients with knee OA. However, considering the potential limitations, more well-performed, large-scale RCTs are needed to verify our findings.

**Supplementary Information:**

The online version contains supplementary material available at 10.1186/s13018-020-02128-0.

## Background

Osteoarthritis (OA) is a debilitating chronic degenerative disease of large joints, especially the hip and knee. The prevalence of symptomatic OA is about 9.6% in men and 18% in women aged 60 years or older in the world [1]. OA is diagnosed by structural abnormalities, such as the loss of articular cartilage, subchondral sclerosis, and marginal osteophyte formation or symptoms associated with these abnormalities, including pain, tenderness, limitations in motion, joint deformity, and instability [2].

Current treatment options for early-stage OA include weight reduction, non-steroidal anti-inflammatory drugs, intra-articular (IA) glucocorticoid injections, and bracing [3, 4]. For end-stage knee OA, total joint arthroplasty is usually used as the mainstay treatment [5]. However, this therapeutic option is associated with serious and life-threatening complications, including an increased risk of infection [5]. Other surgical options include unicompartmental knee arthroplasty (UKA), arthroscopy, and high tibial osteotomy (HTO) [6]. UKA is used as an alternative to total arthroplasty or HTO for single-compartment OA [7]. HTO is a globally recognized treatment option for medial compartment OA of the knee, particularly for patients who are young and active [8].

An alternative treatment for OA of the knee is the IA implantation of mesenchymal stromal cells (MSCs) [9–11]. MSCs were described in the 1970s by Friedenstein et al. [12] and were first tested as a cellular pharmaceutical in human subjects in 1995 by Hillard Lazarus et al. [13]. Since then, they have become the most clinically studied experimental cell therapy platform worldwide [14]. MSCs can repair cartilage [15] and reduce inflammation and pain in the knee, which is due to its anti-inflammatory and immunomodulatory properties [15]. Previous studies have investigated the efficacy and safety of the implantation of MSCs in different OA-affected joints (knee, ankles, or hips), and their results demonstrated the safety and beneficial effects of MSC treatment [16, 17]. Moreover, some other studies suggested that the IA implantation of autologous bone marrow-MSCs in knee OA patients repaired cartilage, ameliorated pain, and improved the quality of life duration a long-term follow-up (1 to 4 years) [18, 19].

There were two published meta-analyses which assessed the efficacy and safety of MSCs used to treat patients with knee OA [9, 11]. One meta-analysis included single-arm studies and quasi-experimental studies [9], and the other included observational studies (case-control, cohort, or comparative study) [11]. The overall quality of the included studies in the previous meta-analysis is poor. Therefore, we performed this updated meta-analysis of randomized control trials (RCTs) to evaluate the efficacy of MSCs in the treatment of patients with knee OA.

## Methods

### Literature search

We conducted this meta-analysis in accordance with the recommendations of the Cochrane Handbook for Systematic Reviews of Interventions and reported it in compliance with the Preferred Reporting Items for Systematic Reviews and Meta-Analyses (PRISMA) statement guidelines (Supplementary file). A comprehensive literature search was performed in the following databases: PubMed, Embase, Web of Science, SinoMed (Chinese BioMedical Literature Service System, China), and CNKI (National Knowledge Infrastructure, China). This literature search was carried out on January 20, 2020, and no language or publication status was imposed. Search terms used was listed as the followings: “cartilage defect,” “cartilage repair,” “osteoarthritis,” “knee osteoarthritis,” “stem cells,” “mesenchymal stem cells” (MSCs), “bone marrow concentrate,” “adipose-derived mesenchymal stem cells” (ADMSCs), “synovial-derived mesenchymal stem cells”, and “peripheral blood-derived mesenchymal stem cells.” In addition, we also searched the reference lists of included studies and relevant reviews in case of the omission of any other potential studies.

### Selection inclusion

Eligible studies were selected based on the following inclusion criteria: (1) study design: RCT; (2) sample size: more than 20; (3) subject: adult patients who had been diagnosed with knee OA by clinical and imaging examination; (4) intervention: MSCs; (5) control: placebo, hyaluronic acid (HA), or other treatment; (6) outcome: McMaster Universities Osteoarthritis Index (WOMAC), visual analog scale (VAS), and International Knee Documentation Committee (IKDC).

### Data extraction

Two independent investigators browsed all the included studies and recorded the features and outcomes of the trial using a data extraction form. The following information of each trial was extracted: first author’s name, year of publication, country, doses of MSCs, sample size, duration of follow-up, control, patients’ baseline characteristics, and the main outcomes (WOMAC, VAS, and IKDC). We also contacted the corresponding authors for missing data when important information was not presented in the original study. Discrepancies between the investigators were resolved by discussion and consensus.

### Risk of bias assessment

We used the method recommended by Cochrane Collaboration to evaluate the risk of bias in each RCT. This method comprised of the following items: random sequence generation, blinding of participants and personnel, blinding of outcome assessment, incomplete outcome data, allocation concealment, selective reporting, and other bias. Based on the assessment rules mentioned above, each RCT was classified as being at high, unclear, or low risk of bias. If an RCT adequately performed the allocation concealment, applied blind for the participants and outcome assessors, and reported the complete outcome data, this RCT was considered to be at low risk of bias. If one or more key domains were not met, this RCT was categorized as a high risk of bias; if one or more key domains were unclear, this RCT was regarded as unclear risk of bias.

### Statistical analysis

We calculated weighted mean difference (WMD) with 95%CI for continuous outcomes. Heterogeneity across studies was evaluated by Cochrane Q and *I*^2^ statistic, in which *P* < 0.1 or *I*^2^ > 50% were considered to be significant [20]. When substantial heterogeneity was identified, a random-effects model (DerSimonian-Laird method) was used to summarize the data; otherwise, a fixed-effects model (Mantel-Haenszel method) was performed. We performed a sensitivity analysis to investigate the influence of excluding any single trial on the overall estimate. Since the number of included studies was less than 10, the assessment of publication bias was not performed. A *P* value less than 0.05 was judged as statistically significant except where a certain *P* value had been given. All analyses were performed using STATA version 12.0 (Stata Corporation, College Station, TX, USA).

### Meta-regression analyses

We hypothesized that differences among the included studies might be influenced by the demographic (mean age and gender) and clinical variables (doses of MSCs and grade of knee OA). In order to explore whether these variables had an impact on the different results across studies, we performed meta-regression analyses after implementing a regression model with WOMAC as a dependent variable(*y*) and the above-mentioned covariates as independent variables (*χ*).

## Results

### Identification of eligible studies

**Fig. 1:**
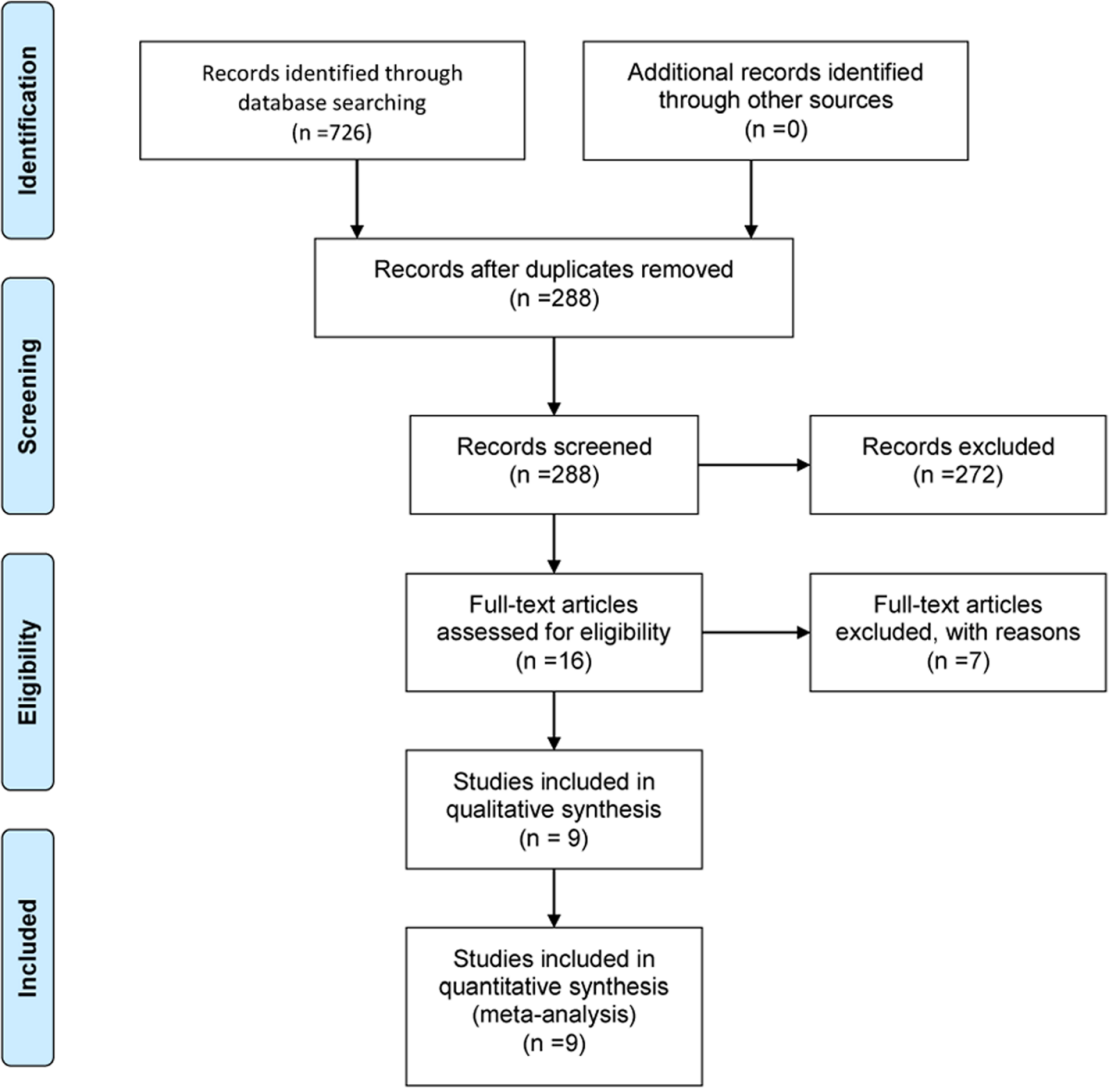
Eligibility of studies for inclusion in the meta-analysis

The flowchart of the literature search process is presented in Fig. 1. The initial search yielded 726 publications, of which 438 were excluded because of duplicate records. The remaining 288 records were left for title/abstract review, among which 272 were excluded because of various reasons (animal experiment, case reports, reviews, or unrelated with our topics). Then, the 16 records were screened for full-text information; however, 7 of them were excluded because of the following reasons: one was a single-arm trial [21], three did not present data of our interest [22–24], and three used MSCs in both groups [16, 25, 26]. Finally, 9 RCTs met the inclusion criteria and were included in this meta-analysis [27–35].

### Study characteristics

The baseline characteristics of the included studies are presented in Table 1. These studies were published between 2010 and 2018. Of these included studies, two were conducted in Spain [28, 30], two in India [29, 31], two in the USA [32, 33], and the remaining three in Iran [27], Singapore [34], and China [35]. The sample size across the included studies ranged from 30 to 87. The duration of follow-up after implantation was also varied in each study, which was 3, 6, 12, 18, and 24 months, respectively. All the patients in the included studies had Kellgren-Lawrence (K-L) radiographic evidence of grade 2 to 4 OA. Of the 9 RCTs, 5 used hyaluronic acid (HA) as control [28, 30, 32, 33, 35], 2 used placebo [27, 29], and the remaining two studies used arthroscopic debridement [31] or HTO [34]. The doses of MSCs varied greatly among the included studies, which ranged from 5 × 10^6^ to 150 × 10^6^. Lamo-Espinosa JM [28] used doses of 10 × 10^6^ and 100 × 10^6^ MSCs; Gupta PK [29] used four different doses of MSCs, including 25 × 10^6^, 50 × 10^6^, 75 × 10^6^, and 150 × 10^6^; Vangsness CT [33] used 50 × 10^6^ and 150 × 10^6^ doses of MSCs. In order to identify whether the doses of MSCs influence the treatment effect, we extracted the data of MSCs in different doses for data analysis.

**Table 1:** Baseline characteristics of patients in the trials included in the meta-analysis

Study	Country	Treatment regimen	No. of patients	Male/female	Age(mean ± SD, year)	Duration of follow-up (month)	K-L criteria (2/3/4)
Emadedin [27]	Iran	40 × 10^6^ MSCs	19	12/7	51.7 ± 9.2	3,6	2/13/4
		Placebo	24	15/9	54.7 ± 5.3	3,6	1/20/3
Lamo-Espinosa [28]	Spain	10 × 10^6^ MSCs	10	4/6	65.9(59.5, 70.6)	6,12	1/2/7
		100 × 10^6^ MSCs	10	8/2	57.8(55.0, 60.8)	6,12	3/3/4
		HA	10	7/3	60.3(55.1, 61.1)	6,12	4/2/4
Gupta [29]	India	25 × 10^6^ MSCs	15	NR	40–70	3,6,12	NR
		50 × 10^6^ MSCs	15	NR	40–70	3,6,12	NR
		75 × 10^6^ MSCs	15	NR	40–70	3,6,12	NR
		150 × 10^6^ MSCs	15	NR	40–70	3,6,12	NR
		Placebo	15	NR	40–70	3,6,12	NR
Vega [30]	Spain	40 × 10^6^ MSCs	15	7/8	57 ± 9	12	NR
		HA	15	6/9	57 ± 9	12	NR
Varma [31]	India	MSCs	25	NR	48.2 ± 5.13	3,6	NR
		Arthroscopic debridement	25	NR	50.67 ± 5.38	3,6	NR
Saw [32]	USA	MSCs+HA	25	10/15	38 ± 7.33	6,12,18	NR
		HA	25	8/17	42 ± 5.91	6,12,18	NR
Vangsness [33]	USA	50 × 10^6^ MSCs	18	13/5	46	6,12,24	NR
		150 × 10^6^ MSCs	18	13/5	46	6,12,24	NR
		HA	19	13/6	46	6,12,24	NR
Wong [34]	Singapore	MSCs+ high tibial osteotomy	28	15/13	53(36–54)	6,12,18,24	NR
		High tibial osteotomy	28	14/14	49(24–54)	6,12,18,24	NR
Ha [35]	China	5 × 10^6^ MSCs	44	14/30	55.6 ± 3.6	3,6,12	10/14/20
		HA	43	12/31	57 ± 3.2	3,6,12	11/11/21

*Abbreviation*: *SD* standard deviation, *K-L* Kellgren-Lawrence, *MSCs* mesenchymal stem cells, *NR* not reported

### Risk of bias assessment

**Fig. 2:**
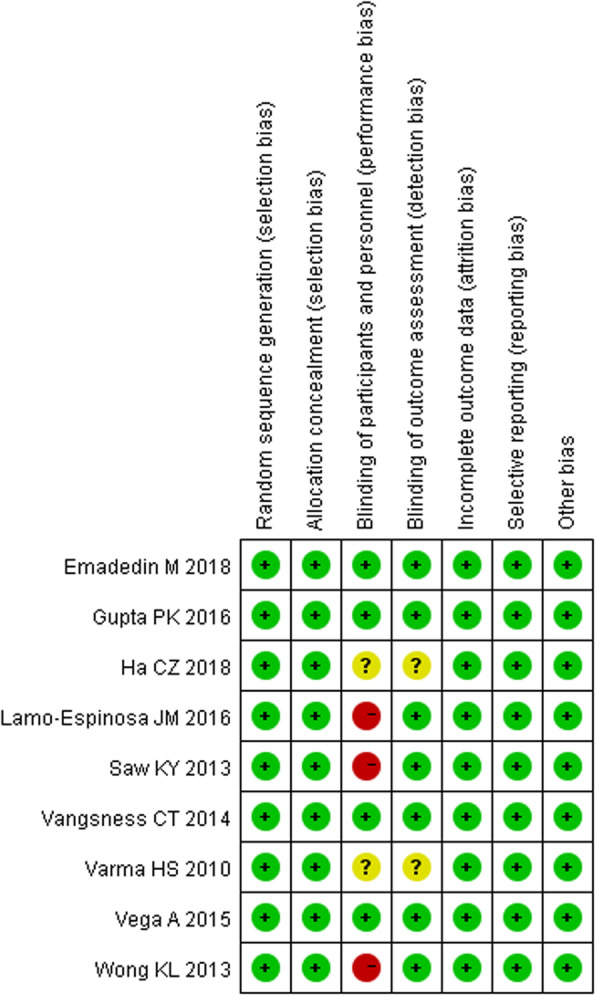
Risk of bias summary

The details of the risk of bias assessment in RCTs are summarized in Fig. 2. Overall, four of the nine included RCTs were classified as being at low risk of bias [27, 29, 30, 33], two at unclear risk of bias [31, 35], and three at high risk of bias [28, 32, 34]. The reason for the trials with unclear risk of bias was that they did not adequately report the performance of binding for participants, personnel, or outcome assessors. The reason for trials with a high risk of bias was that they did not perform the blinding of participants and personnel, which was due to the intervention method or ethical problem.

### VAS score

**Fig. 3:**
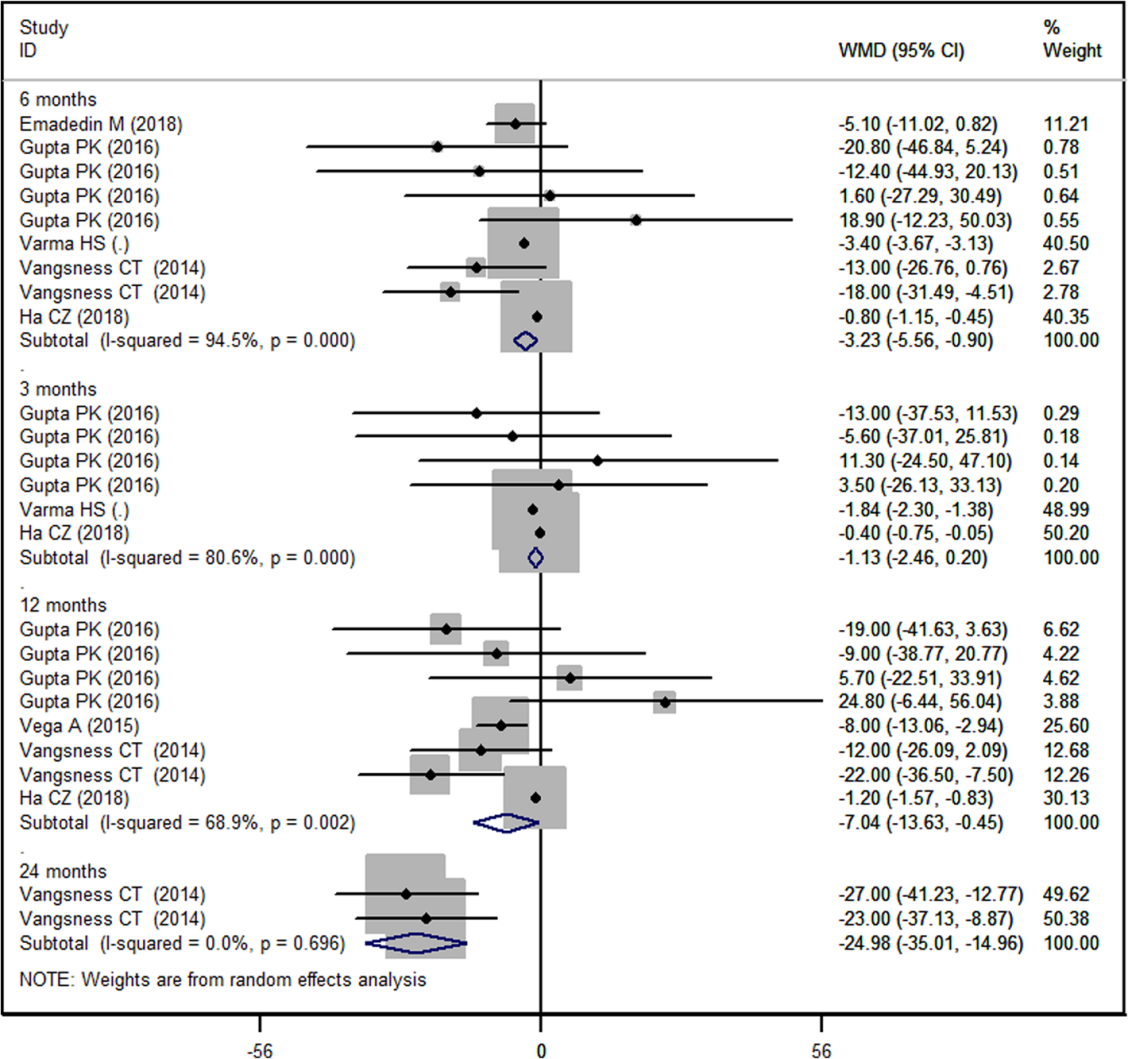
Forest plot showing the effect of MSCs on the VAS score

Six of the included studies reported the data of VAS [27, 29–31, 33, 35]. The VAS score decreased from baseline in both MSC and control groups. The aggregated data suggested that the MSCs was associated with a significantly greater reduction in VAS score than control, and this difference was seen at 6 months, 12 moths, and 24 months, but not at 3 months (3 month: WMD = − 1.13, 95%CI: − 2.46, 0.20, *P* = 0.097; 6-month: WMD = − 3.23, 95%CI: − 5.56, − 0.90, *P* = 0.007; 12-month: WMD = − 7.04, 95%CI: − 13.63, − 0.45, *P* = 0.036; 24-month: WMD = − 24.98, 95%CI: − 35.01, − 14.96, *P* < 0.001) (Fig. 3).

### WOMAC pain score

**Fig. 4:**
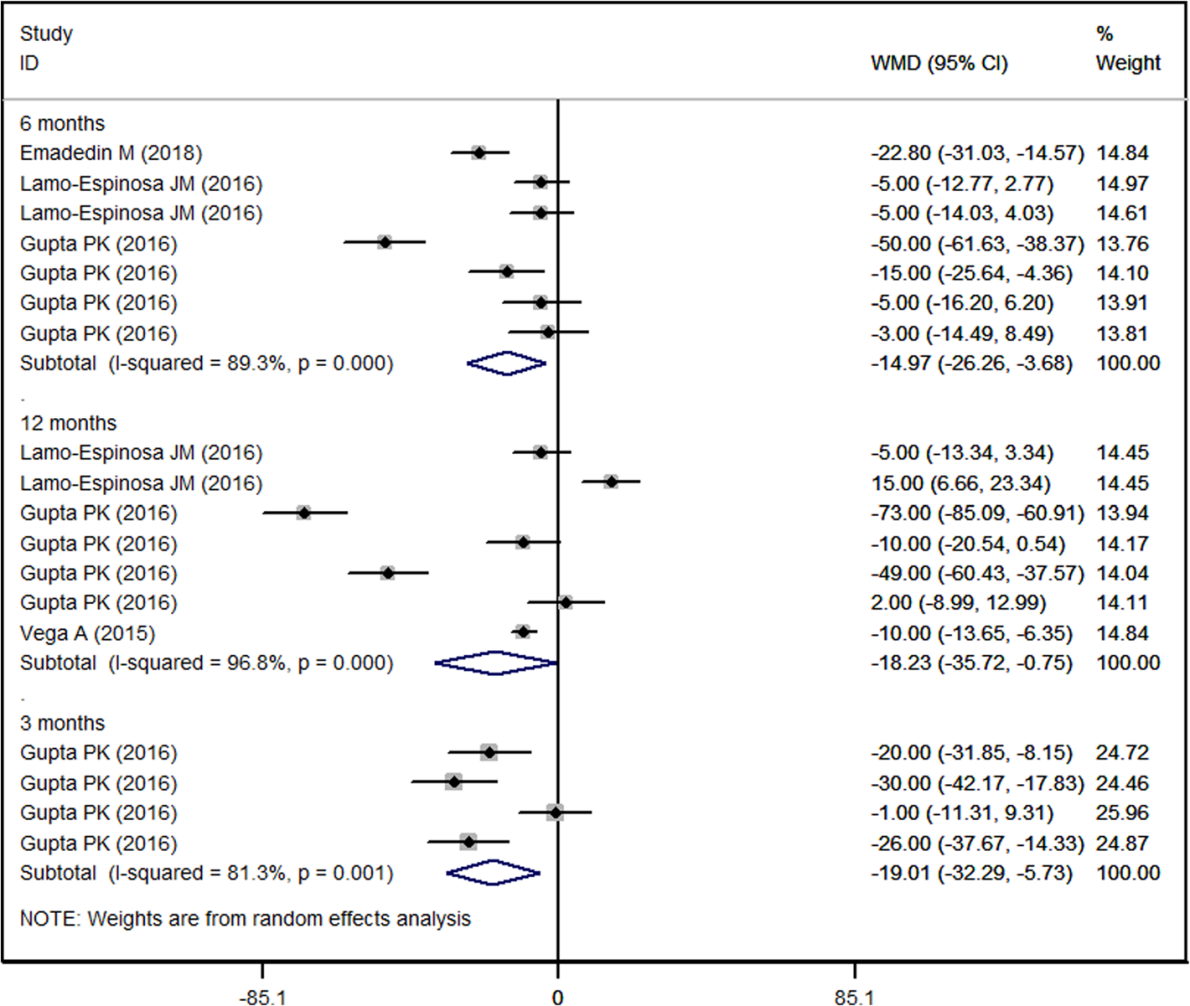
Forest plot showing the effect of MSCs on the WOMAC pain score

Four of the included studies reported the data of WOMAC pain [27–30]. The WOMAC pain score was decreased in both MSC and the control group. Pooled data showed that the reduction in WOMAC pain score was significantly greater in the MSC group than in control group, and this difference was seen through all the study period (3-month: WMD = − 19.01, 95%CI: − 32.29, − 5.73, *P* = 0.005; 6-month: WMD = − 14.97, 95%CI: − 26.26, − 3.68, *P* = 0.009; 12-month: WMD = − 18.23, 95%CI: − 35.72, − 0.75, *P* = 0.041) (Fig. 4).

### WOMAC stiffness score

**Fig. 5:**
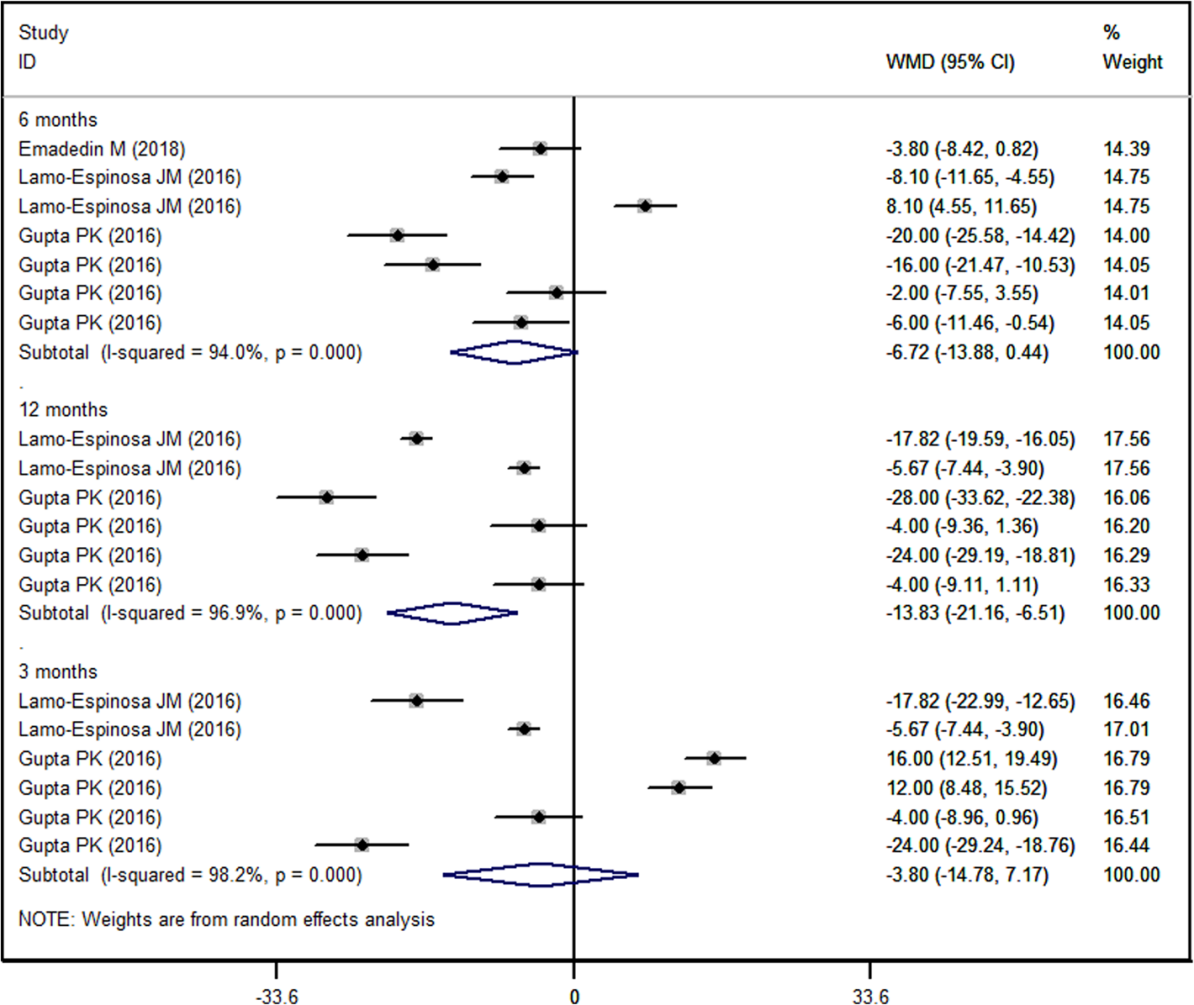
Forest plot showing the effect of MSCs on the WOMAC stiffness score

Three of the included studies reported the data of WOMAC stiffness [27–29]. The WOMAC stiffness score was reduced in both MSC and control groups. Pooled data showed that the reduction associated with MSCs in WOMAC stiffness was significantly greater than that in control. This difference was only observed at 6 months and 12 months, but not at 3 months (3-month: WMD = − 3.80, 95%CI: − 14.78, 7.17, *P* = 0.497; 6-month: WMD = − 6.72, 95%CI: − 13.88, 0.44, *P* = 0.066; 12-month (WMD = − 13.83, 95%CI: − 21.16, − 6.51, *P* < 0.001) (Fig. 5).

### WOMAC function score

Three of the included studies reported the data of the WOMAC function score [27–29]. Both MSCs and control resulted in a reduction in the WOMAC function score. Pooled results indicated that the reduction in WOMAC function score was significantly higher in the MSC group than that in the control group, and this difference was seen through all the treatment period (3-month: WMD = − 8.14, 95%CI: − 15.34, − 0.95, *P* = 0.027; 6-month: WMD = − 7.61, 95%CI: − 13.62, − 1.61, *P* = 0.013; 12-month: WMD = − 8.32, 95%CI: − 15.63, − 1.01, *P* = 0.026).

### WOMAC total score

Three of the included studies reported the data of WOMAC total score [27, 28, 30]. The WOMAC total score was reduced in both MSC and control groups. However, the reduction between them was not significantly different, and this was consistent throughout the treatment period (3-month: WMD = 3.35, 95%CI: 0.01, 6.69, *P* = 0.049; 6-month: WMD = − 4.57, 95%CI: − 25.28, 16.13, *P* = 0.665; 12-month: WMD = − 1.56, 95%CI: − 9.78, 6.66, *P* = 0.710).

### IKDC score

Two of the included studies reported the data of the IKDC score [32, 34]. The IKDC score was improved in both MSC and control groups. Pooled data showed that the improvement between the two groups was not significantly different, and this was consistent throughout the treatment period (6-month: WMD = 2.22, 95%CI: − 5.61, 10.04, *P* = 0.579; 12-month: WMD = 3.92, 95%CI: − 6.49, 14.33, *P* = 0.461; 18-month: WMD = 6.71, 95%CI: − 7.74, 21.15, *P* = 0.363; 24-month: WMD = 5.24, 95%CI: − 0.92, 11.40, *P* = 0.095).

### Meta-regression

Table 2 showed the results of a multivariate meta-regression for demographic (mean age and gender) and clinical variables (doses of MSCs and OA grade). Due to the limited number of studies with sufficient information on these variables, meta-regression was calculated for the VAS score only. Results indicated that none of these variables significantly predicted the effects of MSCs on the VAS score.

**Table 2:** Results of multivariate meta-regression analyses for demographic (mean age and gender) and clinical variables (doses of MSCs and OA grade) to predict effects of MSCs on the VAS score

Covariate	Coefficient	95%CI	*t* value	*P* value
Age	0.4879	− 0.3639, 1.3398	1.19	0.248
Gender	0.4929	− 0.3598, 1.3457	1.20	0.244
Grade of osteoarthritis	0.5032	− 0.3489, 1.3555	1.22	0.234
Duration of disease	0.5177	− 0.3416, 1.3769	1.25	0.225
Dose of MSCs	0.4893	− 0.3635, 1.3421	1.19	0.247

*Abbreviations*: *MSCs* mesenchymal stem cells, *OA* osteoarthritis

## Discussion

This is a further meta-analysis to evaluate the efficacy of MSC therapy in patients with knee OA. Based on 9 RCTs, our results had greater power to assess the effect of MSCs in the treatment of patients with knee OA. Results from our study suggested that the use of MSCs significantly reduced the pain and improved stiffness and function in the long term. The present study indicated that MSC therapy could be used as a potentially efficacious treatment for knee OA.

There have been several published meta-analyses of MSC therapy in the treatment of patients with knee OA [9, 11, 36]. Our study expands on the previous meta-analysis to provide better evidence for the effect of MSCs. First, the quality of the included studies in this meta-analysis was higher than that in the previous meta-analysis. Cui GH et al. [9] performed a systematic review of 18 clinical trials published before December 2014 to assess the clinical efficacy and safety of MSCs for knee OA. However, among the included studies, 10 were single-arm studies, 4 were quasi-experimental studies, and 4 were RCTs. Single-arm studies were unable to provide available data for estimate pooling since they were lack of control. Quasi-experimental studies could not exclude the influence of confounding factors on the treatment effect since they were carried out without random allocation design. These studies mentioned above had their inherent weakness that might influence the final overall estimate, and it was not appropriate to include them for data analysis. Ma YB et al. [11] performed another meta-analysis of 11 studies to evaluate the therapeutic efficacy and safety of MSCs for patients with knee OA. One of the inclusion criteria was that the eligible study must be an RCT. However, among the 11 included studies, only 4 were RCTs, and the remaining 7 were case-control or comparative studies. These observational studies did not meet the inclusion criteria.

Moreover, they were more likely to result in selection bias or information bias. Thus, the pooled results based on data from these studies were not reliable. Whereas in this meta-analysis, all the included studies were performed with an RCT design, and four of them were classified as being at a low risk of bias, which added the robustness of our results. Second, although all the included studies in this meta-analysis were RCTs, and most of them had a low risk of bias, we could not exclude the influence of demographic and clinical variables on the overall estimate. Thus, we performed a meta-regression analysis to investigate the impacts of these variables on the VAS score, which had not been conducted in the previous meta-analysis.

In the present study, we found that the MSCs significantly relieve the pain and improved the function and stiffness in patients with knee OA. Our results were consistent with the findings of previous studies [27, 30]. Lamo-Espinosa et al. [27] performed a randomized, triple-blind, and placebo-control of IA implantation of MSCs used in knee OA patients. In that study, 43 patients (K-L grades 2, 3, and 4) were randomly assigned into either the MSCs (40 × 10^6^ cells) or normal saline (placebo) groups. The WOMAC pain score at the end of 6 months was significantly improved in the MSC group than that in the placebo group (SE = − 21.8, 95%CI: − 33.8, − 9.9) [27]. Whereas some other studies reported different results [29, 36], Xia et al. included seven RCTs or controlled clinical trials to assess the effects of MSCs in patients with knee OA. Their results suggested that MSC treatment did not reduce the pain (WMD = − 1.33, 95%CI: − 3.08, 0.41; *P* = 0.13) [36]. However, results from two high-quality trials observed positive results, which suggested that the WOMAC pain was significantly improved with the treatment of MSCs (WMD = − 0.49, 95%CI: − 0.79, − 0.19; *P* = 0.001) [36]. Even so, the authors did not draw solid conclusions, since the included studies had substantial differences in study design, cell production methods, and dosage [36].

Another randomized, double-blind, multicentric, placebo-controlled, and phase 2 trial [29] also suggested that MSCs had no effect on the pain relief. Gupta et al. [29] performed this trial in 60 OA patients, who were randomized to receive different doses of cells (25 × 10^6^, 50 × 10^6^, 75 × 10^6^, or 150 × 10^6^ cells) or placebo. They were evaluated by using VA and WOMAC index at 1, 3, 6, and 12 months of follow-up for cartilage evaluation. Among these patients, those who were treated with 25 × 10^6^ MSCs achieved a trend towards improvement in the WOMAC pain score as compared with those treated with placebo; however, this difference was not statistically significant [29]. It should be noted that, in the preclinical model of that study, the pain score in high-dose animals was continued to improve until the end of the study (12 weeks) [29]. Although the exact mechanism behind the effect of MSCs on pain relief is not uncertain, several studies have suggested that the anti-inflammatory activity is responsible for the effect [37–39]. In animal studies, it has been reported that the increased levels of pro-inflammatory cytokines might result in the pain increase. The IA implantation of MSCs plays a crucial role in pain relief by secreting many kinds of anti-inflammatory cytokines and analgesic peptides [40].

The treatment dose is one of the most important factors that influence the treatment effect of MSCs. However, the optimal dose remained unclear among the previous studies. Gupta et al. [29] found that the low dose of MSCs (25 × 10^6^ cells) significantly improved the pain measurement scores, whereas the high dose (50 × 10^6^, 75 × 10^6^, or 150 × 10^6^ cells) did not. In that study, the VAS and WOMAC composite index scores at the 12-month reduced by 64% and 64.4% in the 25 × 10^6^ dose of MSC group, as compared with 36% and 49.3% in the control group, respectively [29]. The authors speculated that the following reasons might explain the beneficial effect of low-dose MSCs: first, in that study, HA (2 ml) was used as the supporting matrix, and the dose of 25 × 10^6^ cells might be optimum with the volume of HA. Second, in the limited intra-articular space of the knee joint, the dose of 25 × 10^6^ cells might be optimal. Third, because of the high cell concentration or limited space in the knee joint, the doses higher than 25 × 10^6^ cells might result in cell aggregation, which subsequently causes cell death. Fourth, the dose of 25 × 10^6^ cells might lie at the upper limit of the efficacy dose, since the doses of (10–25) × 10^6^ cells demonstrate efficacy in the treatment of knee OA [17, 34, 41, 42]. Finally, the higher dose of MSCs might stimulate MSCs to M1-type cells, which are involved in the pro-inflammatory response, whereas the dose of 25 × 10^6^ cells might activate the MSCs into M2-type cells, which are involved in the anti-inflammatory/immunosuppressive response [43]. In the present study, due to the doses of MSCs varied greatly among the included studies, we did not perform subgroup analysis to identify the optimal treatment dose. Further dose-finding studies are required to explore which cell dose would result in the best outcome.

The method of delivery of cells is another important factor that influences the treatment efficacy. In the study of Wakitani et al. [44], adherent cells of bone marrow embedded in collagen gel were transplanted into the articular cartilage defect in patients who underwent HTO. In Korea, a combination production, Cartistem ®, has been used in the damaged area by arthroscopy after performing a microfracture [45]. These open surgical methods might extend hospital length of stay and increase the health care cost, as well as the pain. Compared with the open surgical approach, minimally invasive techniques, such as IA injection, have the following advantages: less invasive, decreased hospital stay, and ease of implementation in larger patients. Moreover, ultrasound guidance of knee injections, as a better method, could deliver the cells precisely. Previous study has revealed that the IA accuracy of needle placement in ultrasound guidance of knee injection group (95.8%) was significantly higher than that in anatomical guidance group (77.8%) [46]. Thus, the ultrasound-guided knee injection can improve the clinical outcomes to patients.

There were several potential sources in this study, which should be taken into account when interpreting our results. First, we found substantial heterogeneity across the included studies. Although we performed meta-regression to explore the potential sources of heterogeneity, no significant information was found. However, one should not be surprising given the differences in doses of MSCs, coadjuvants, and cell processing methods among the included studies. These factors might account for heterogeneity and influenced the overall estimates. Second, this meta-analysis was performed on 9 RCTs, and three of them had a relatively small sample size. This might lead to result bias since smaller trials were more inclined to overestimate the treatment effect as compared to larger trials. Third, due to the insufficient data of included studies, we did not perform dose-response analysis to identify the optimal treatment dose of MSCs.

## Conclusion

In conclusion, this study indicated that MSC therapy significantly reduced the pain, stiffness, and function in patients with knee OA as compared with control. This treatment effect could last for a long term of 24 months. Thus, MSC therapy can be used as a potentially efficacious treatment for knee OA. However, considering the limitations of this study, well-performed, larger-scale RCTs are needed to verify our findings.

## Supplementary Information

**Additional file 1.**

## Data Availability

The datasets used and/or analyzed during the current study are available from the corresponding author on reasonable request.

## Abbreviations

MSCs: Mesenchymal stromal cells
OA: Osteoarthritis
RCTs: Randomized controlled trials
VAS: Visual analog scale
WOMAC: McMaster Universities Osteoarthritis Index
IKDC: International Knee Documentation Committee
WMD: Weight mean difference
